# Invasive Lobular Carcinoma of the Breast With Rectal Metastasis: A Rare Case Report

**DOI:** 10.7759/cureus.23666

**Published:** 2022-03-30

**Authors:** Nada E Algethami, Amjad A Althagafi, Rawan A Aloufi, Fawaz A Al Thobaiti, Hamma A Abdelaziz

**Affiliations:** 1 Medicine, Taif University, Taif, SAU; 2 General Surgery, Al Hada Armed Forces Hospital, Taif, SAU; 3 General and Colorectal Surgery, Al Hada Armed Forces Hospital, Taif, SAU

**Keywords:** metastases to colorectum, secondary tumor, malignancy, rectal metastasis, breast metastasis

## Abstract

The rectum is a relatively unusual site for metastasis from invasive lobular carcinoma (ILC) of the breast, and it carries dangers such as perforation and blockage. We reported a case of a 47-year-old female patient complaining of breast ILC for one year. Recently, the patient complained of abdominal distention, mild generalized abdominal pain, and weight loss. The abdominal ultrasound (US) showed moderate ascites without hepatomegaly, and ascitic tapping was positive for malignant cells. Lower colonoscopy showed a congested mass of 8 cm, and anal verge biopsy showed colonic mucosa laminal propria infiltrated with atypical cells and adenocarcinoma metastatic from the breast. In a patient with breast cancer, particularly ILC, who has developed new gastrointestinal tract (GIT) symptoms, there is significantly a high chance of rectal metastatic illness. Early detection is critical for successful treatment.

## Introduction

Breast cancer is the most frequent malignancy worldwide and one of the leading causes of cancer-related death in women aged 20-59 years [1,2]. In Saudi Arabia, the prevalence rate of breast cancer is 53% [3]. Invasive lobular carcinoma (ILC) represents approximately 10% of all invasive breast cancers, and it is the second most common subtype of breast cancer [4]. ILC is diagnosed at the mean age of 57 years [5]. The risk factors of ILC do not differ from those of other types of breast cancer, are hormone-dependent, and include the age of menopause, age at menarche, nulliparity, age of first birth, and obesity [6]. Some women have a clearly palpable lump, whereas others have diffuse nodularity, have vague skin thickening, or are clinically occult [6]. The diagnostic process of ILC is similar to that of other types of breast cancer and includes ultrasonography, mammography, and magnetic resonance imaging if indicated [1].

Diagnosing ILC by a mammogram is challenging due to the subtle changes that may mimic normal breast parenchyma [7]. Unfortunately, 30% of breast tumors will progress to stage IV following treatment, and 5%-10% will be metastatic at the time of diagnosis [8]. ILC frequently affects the lungs, bones, reproductive organs, central nervous system, gastrointestinal tract (GIT), peritoneum, and retroperitoneum [9]. Metastasis from the breast to GIT is uncommon in clinical practice; however, it has been reported in autopsy series ranging from 8% to 35% [10]. Overall, upper and lower GIT metastases account for 0.03% and 0.05% of all metastases, respectively [11]. Metastasis to the rectum is a particularly rare site and carries risks such as perforation or obstruction [12,13]. In this article, we present a case of a 47-year-old woman diagnosed with metastatic ILC in the rectum after five years.

## Case presentation

A 47-year-old female patient with hypothyroidism who complained of a left painless breast mass for one year appeared during her last pregnancy. She was lactating and had no nipple discharge, history of oral contraceptive (OCP), or family history of breast cancer. On examination, left breast deformity with tethering skin was identified. There was an ill-defined mass (6 cm × 8 cm) in the left upper lateral quadrant at 1 o'clock fixed to the skin but not to the chest wall; there was no nipple discharge, but there was a palpable left axillary lymph node.

Ultrasound (US) breast examination was performed, which showed homogenous breast echotexture with a hypoechoic focal lesion in the left breast at the 1 o'clock position, measuring approximately 4.3 cm × 3.7 cm with irregular outline and posterior shadowing. Biopsy was performed, which showed ILC ER++ (estrogen receptor positive), PR +ve (progesterone receptor positive), and HER2 -ve (human epidermal growth factor receptor 2 negative); a dynamic MRI study of both breasts revealed dense, heterogeneously enhanced parenchyma in both breasts. The left breast showed an iII-defined heterogeneously enhanced mass lesion at the upper outer part. Its dynamic curve showed a moderate uptake of contrast followed by a plateau phase (type Il curve) with a late washout.

Irregular spiculation was observed at the outer part of the overlying skin. A malignant lesion was suggested for histopathological correlation. The right breast showed heterogeneous enhancement without a sizable three-dimensional mass lesion in the outer half of the breast parenchyma. However, this heterogeneous enhancement of the parenchyma that appears partially nodular and partially patchy shows a type II dynamic curve in the dynamic contrast study. One of these nodular shadows may represent a small fibroadenoma previously observed by the US. For the right breast and within average echogenicity of the scanned breast, an oval-shaped hypoechoic lesion that was horizontally oriented without surrounding parenchymal invasion was observed. The lesion measured 7 mm × 3 mm and was a small fibroadenoma at the 10 o'clock position. Another small cyst (approximately 5 mm × 2 mm) was observed at the 12 o'clock position. There were prominent retro-areolar ducts without signs of parenchymal invasion or distortion. Enlarged axillary lymph nodes were observed inside the right axilla with intact fatty hilum, which suggested their benign nature, and breast imaging-reporting and data system II (BIRADS II) of the right breast was suggested. CT chest, abdomen, and pelvis (CT-CAP) -ve for metastasis, negative bone scan, and patient counseling were performed prior to lift modified radical mastectomy (MRM) with right breast biopsy instead of right breast prophylactic mastectomy. The patient underwent lift MRM and right prophylactic mastectomy. During final histopathology, bilateral multicentric ILC was detected.

The case was discussed in the tumor board, which recommended right axillary dissection; the patient underwent lymphadenectomy, optional hormonal therapy + radiotherapy, but not chemotherapy because of ER + PR +ve and Ki67 score of 1%. The patient refused four cycles of chemotherapy, accepted hormonal therapy of tamoxifen 20 mg once daily (OD), refused ovary suppression, and accepted radiotherapy RTx 27 in January 2018. The oncology course was followed by obstetricians and gynecologists for bilateral cysts. In August 2019, the patient was TAH + BSO (total abdominal hysterectomy + bilateral salpingo-oophorectomy) cancer-free and was on letrozole, and the dual-energy x-ray absorptiometry (DEXA) scan showed osteopenia. In June 2021, she had neoplastic ascites and was started on faslodex and palbociclib.

In October 2021, an x-ray of the chest revealed left-sided pleural effusion and left lower lobe consolidation. The patient complained of abdominal distension with mild generalized abdominal pain with significant weight loss. Abdominal US report showed moderate ascites without hepatomegaly. The patient underwent ascitic tapping, and the material was sent for cytology and came back positive for malignant cells. For ascites, a pig tube was inserted, and 5 L of fluid was collected in 1.5 h. The patient’s abdominal pain did not improve, and the patient was referred to a gastroenterologist. The gastroenterologist performed an upper and lower gastrointestinal examination. Upper endoscopy showed gastritis; biopsy was performed, which came back positive for *Helicobacter pylori*.

**Figure 1:**
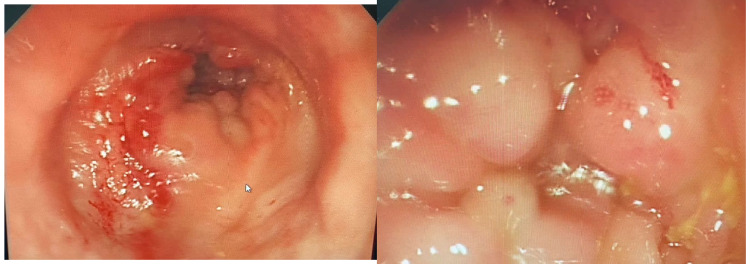
Lower colonoscopy showing an 8-cm congested mass The image shows an ugly-looking mass that bleeds easily if touched with the scope and involves all the circumferences with pending obstruction.

**Figure 2:**
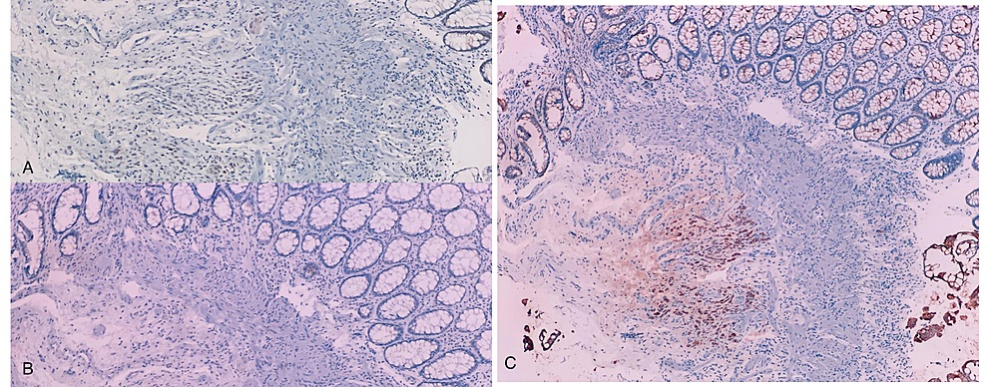
Immunohistochemical stain (A and B) ER and PR positivity confirms the breast origin. (C) Pan-CK immunohistochemical stain positivity confirms the epithelial origin. ER: Estrogen receptor; PR: Progesterone receptor; Pan-CK: Pan-cytokeratin.

The lower colonoscopy showed an 8-cm congested mass, which was about 4 cm from the anal verge (Figure 1). Anal verge biopsy showed colonic mucosa lamina propria infiltrated with two atypical cells, and the adenocarcinoma metastatic from the breast with immunohistochemical stain indicated ER++, HER2+, and Pan-cytokeratin (Pan-CK+++) (Figure 2).

## Discussion

Breast cancer is the most recorded malignancy among females and is the main cause of morbidity and mortality [14]. The most common primary tumor that metastasizes to the GIT is breast carcinoma. Interestingly, lobular carcinoma which accounts for only 8%-14% of all breast adenocarcinomas rather than the more prevalent invasive ductal carcinoma is the most common source of extrahepatic GIT metastatic illness from breast carcinoma [15,16]. This may be related to the proclivity of lobular cells for GIT [15,16]. Approximately, 60% of cases with breast cancer had distant metastases at the time of diagnosis, whereas 30%-80% may experience metastatic disease after cancer surgical intervention, radiotherapy, or endocrine therapy [17].

Breast cancer mostly metastasizes to bone, lymph nodes, lung, liver, or brain. GIT metastasis is rare [18], and the most reported GIT sites of metastases are the stomach and small intestine [19,20]. Several studies, including case reports, reported colorectal metastatic due to primary lobular carcinoma of the breast [21,22]. Other studies focused on the clinical or radiological variety of metastatic breast lobular carcinoma of the colorectum [23-25].

Winston et al. found that GIT metastasis from primary breast cancer was as rare as 73 cases out of 12,001 reported metastatic cases of which only 24 cases had colorectal metastasis [26]. The histological assessment showed that lobular carcinoma of the breast prevailed compared to ductal carcinoma [27]. Recently, in 2018, Ruymbeke et al. reported a 65-year-old woman who was diagnosed with infiltrative lobular carcinoma of the left breast with bone metastases and developed metastasis of the rectum and anal canal four years later [28]. A focused literature search revealed only six cases of anal metastases, three with ILC and three with invasive ductal carcinoma [28-33]. The rarity of this event, remarkably long interval, and non-specific clinical presentation make it difficult to diagnose GIT metastasis from primary breast carcinoma. Early diagnosis and correct recognition are essential for an acceptable therapeutic approach [10].

Szabó et al. determined that metastasis of lobular carcinoma of the breast to the colon mimicked inflammatory bowel disease including Crohn’s disease [34]. In addition, Koos et al. reported a case with multiple metastases simulating Crohn's disease both radiologically and intra-operatively in the colon and small bowel [35]. When metastatic cancer remains in the submucosa, it is challenging to endoscopically obtain satisfactory biopsy samples [34].

## Conclusions

This case report declares the significance of a high likelihood of clinical prediction of rectal metastatic disease in a patient with breast malignancy (especially ILC) with new GIT symptoms. Clinical, endoscopic, and radiological presentations may vary and may have non-specific and confusing features. Early and proper diagnosis is vital for satisfactory treatment. As GIT involvement is mostly seen in extensive metastatic disease, the prognosis is still mostly unsatisfactory.
